# A Multi-targeted Drug Candidate with Dual Anti-HIV and Anti-HSV Activity

**DOI:** 10.1371/journal.ppat.1003456

**Published:** 2013-07-25

**Authors:** Jan Balzarini, Graciela Andrei, Emanuela Balestra, Dana Huskens, Christophe Vanpouille, Andrea Introini, Sonia Zicari, Sandra Liekens, Robert Snoeck, Antonín Holý, Carlo-Federico Perno, Leonid Margolis, Dominique Schols

**Affiliations:** 1 Rega Institute for Medical Research, KU Leuven, Leuven, Belgium; 2 Department of Experimental Medicine and Biochemical Science, University of Roma Tor Vergata, Rome, Italy; 3 Program of Physical Biology, Eunice Kennedy-Shriver National Institute of Child Health and Human Development, National Institutes of Health, Bethesda, Maryland, United States of America; 4 Institute of Organic Chemistry and Biochemistry, Academy of Sciences of the Czech Republic, Prague, Czech Republic; Emory University, United States of America

## Abstract

Human immunodeficiency virus (HIV) infection is often accompanied by infection with other pathogens, in particular herpes simplex virus type 2 (HSV-2). The resulting coinfection is involved in a vicious circle of mutual facilitations. Therefore, an important task is to develop a compound that is highly potent against both viruses to suppress their transmission and replication. Here, we report on the discovery of such a compound, designated PMEO-DAPym. We compared its properties with those of the structurally related and clinically used acyclic nucleoside phosphonates (ANPs) tenofovir and adefovir. We demonstrated the potent anti-HIV and -HSV activity of this drug in a diverse set of clinically relevant *in vitro*, *ex vivo*, and *in vivo* systems including (i) CD4^+^ T-lymphocyte (CEM) cell cultures, (ii) embryonic lung (HEL) cell cultures, (iii) organotypic epithelial raft cultures of primary human keratinocytes (PHKs), (iv) primary human monocyte/macrophage (M/M) cell cultures, (v) human *ex vivo* lymphoid tissue, and (vi) athymic nude mice. Upon conversion to its diphosphate metabolite, PMEO-DAPym markedly inhibits both HIV-1 reverse transcriptase (RT) and HSV DNA polymerase. However, in striking contrast to tenofovir and adefovir, it also acts as an efficient immunomodulator, inducing β-chemokines in PBMC cultures, in particular the CCR5 agonists MIP-1β, MIP-1α and RANTES but not the CXCR4 agonist SDF-1, without the need to be intracellularly metabolized. Such specific β-chemokine upregulation required new mRNA synthesis. The upregulation of β-chemokines was shown to be associated with a pronounced downmodulation of the HIV-1 coreceptor CCR5 which may result in prevention of HIV entry. PMEO-DAPym belongs conceptually to a new class of efficient multitargeted antivirals for concomitant dual-viral (HSV/HIV) infection therapy through inhibition of virus-specific pathways (i.e. the viral polymerases) and HIV transmission prevention through interference with host pathways (i.e. CCR5 receptor down regulation).

## Author Summary

To contain the HIV-1 epidemic, it is necessary to develop antivirals that prevent HIV-1 transmission. It is well known that HIV infection might be accompanied by other pathogens, which often are engaged with HIV-1 in a vicious circle of mutual facilitation. One of the most common of these pathogens is herpes simplex virus (HSV) type 2. Since there is an urgent need for a next generation antivirals that are multi-targeted, we can now report on the development of the first antiviral of this new generation that efficiently suppresses both HIV-1 and HSV-2. We found that the dual-targeted antiviral drug affects several targets for viral replication. It uniquely combines in one molecule three important abilities: (i) to efficiently suppress HSV-encoded DNA polymerase, (ii) to efficiently suppress HIV-1-encoded reverse transcriptase, and (iii) to stimulate secretion of CC-chemokines that downregulate the HIV-1 coreceptor CCR5. The compound suppresses both viruses in a wide-range of in vitro, ex vivo, and in vivo experimental models. The ability of one molecule to suppress HIV-1 and HSV-2 by combining direct activity against their two key enzymes and indirect immunomodulatory effects is unique in the antiviral field.

## Introduction

**Figure 1:**
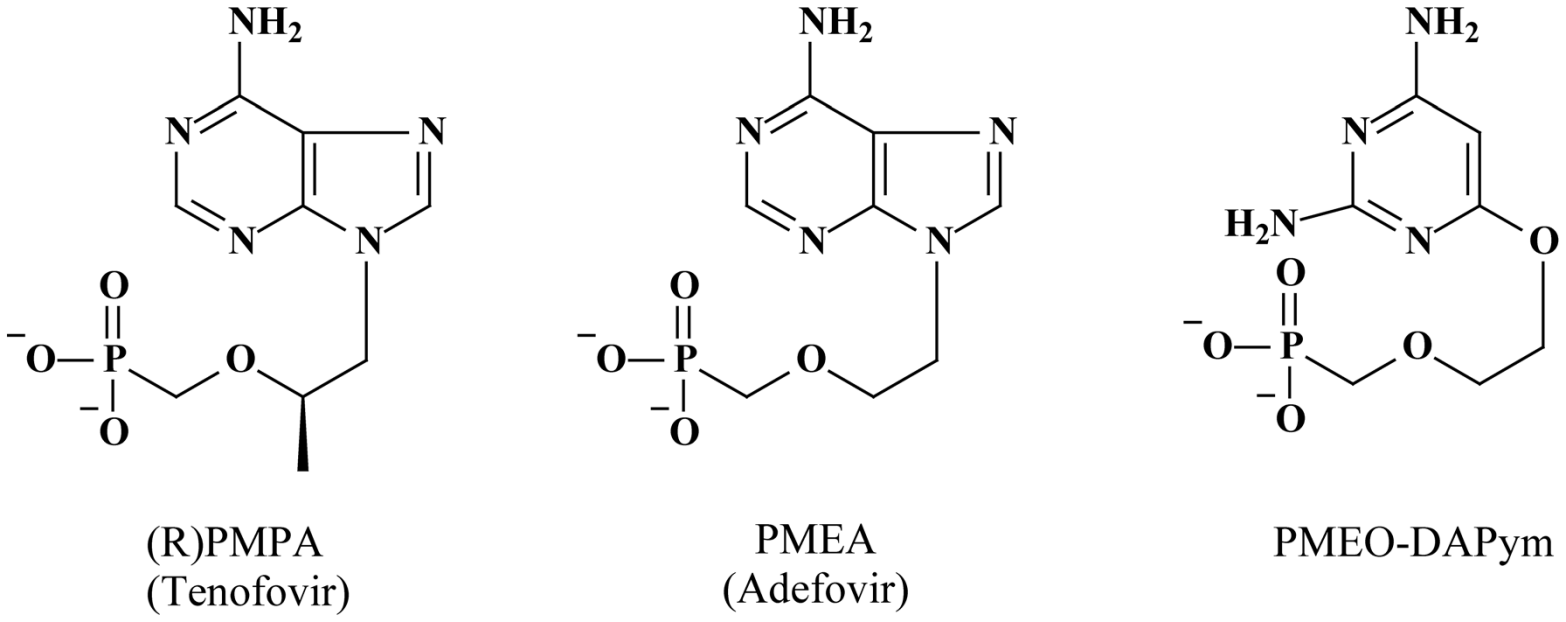
Structural formulae of the acyclic nucleoside phosphonates. Adefovir and tenofovir are purine (adenine) analogues. PMEO-DAPym is a (2,4-diamino)pyrimidine analogue [46], [47]. Molecular modeling revealed that the PMEO-derivatives are structural mimics of the corresponding purine (2-aminoadenine) analogues [53], and it was recently shown that PMEO-DAPym also functionally behaves as an adenine analogue like adefovir and tenofovir [54].

Human immunodeficiency virus (HIV) infection is commonly associated with other sexually transmitted infections such as herpes simplex virus type 2 (HSV-2). Such infections with HSV-2 may facilitate the risk of HIV acquisition and often worsen the clinical course of the HIV disease [1]–[5]. In fact, HIV-1 has been recovered frequently from genital herpes lesions in co-infected individuals [6]. Although HSV target cells in tissues are still poorly understood and it is not known whether macrophages are important targets for HSV *in vivo*, both HIV-1 and HSV-2 can infect macrophages. Cells of the monocyte/macrophage (M/M) lineage reside in genital mucosal tissues and are thought to be reservoirs of HIV-1 in the genital tract [7], [8]. Also, there is evidence that HSV infection can also stimulate macrophages *in vitro* and induce HIV-1 replication in these cells [9]. Thus, it would be beneficial if a microbicide has efficient suppressive activity against both HIV-1 and HSV-2. Highly specific drugs, such as the acyclic nucleoside phosphonate (ANP) analogue 9-(2-phosphonylmethoxypropyl)adenine [(*R*)PMPA; tenofovir] against HIV [10] and the nucleoside analogue 9-(2-hydroxyethyloxymethyl)guanine (acyclovir; ACV) against HSV [11], have been developed. Unexpectedly, treatment of HIV-1/HSV-2–coinfected individuals with acyclovir diminishes both HSV-2 and HIV-1 load [12]–[15], while topically applied tenofovir diminishes transmission not only of HIV-1 [16] but also of HSV-2 [17]. Both drugs have been found to be directly active against these two viruses [18], [19], although suppression of HIV-1 by acyclovir and of HSV-2 by tenofovir is suboptimal. It would be advisable if antiviral agents can be designed and developed that concomitantly display pronounced inhibitory activity against both pathogens. Therefore, the different subclasses of acyclic nucleoside phosphonates were revisited because it has been previously shown that several members of the ANPs display potent activity against a variety of DNA viruses, including herpesviruses and hepatitis B virus, and retroviruses [20]–[22]. From a wide screen of hundreds of ANPs, a compound designated 6-phosphonylmethoxyethoxy-2,4-diaminopyrimidine (PMEO-DAPym) (Fig. 1) emerged as a novel prototype drug that concomitantly act as an efficient inhibitor of both HIV-1 and HSV-2 replication, but that was also surprisingly endowed with a capacity to interact with HIV entry. This combination of unique properties in one single molecule makes it a promising new-generation multitargeted antiviral for dual-viral (HSV/HIV) infection therapy and HIV transmission prevention.

## Results

### Anti-HIV-1 and anti-HSV-2 activity in laboratory cell cultures

We compared the activity of PMEO-DAPym with those of tenofovir (Fig. 1), a commonly used anti-HIV nucleotide reverse transcriptase (RT) inhibitor (NtRTI) that also shows anti-HSV-2 activity [10], [19] and 9-(2-phosphonylmethoxyethyl)adenine (PMEA; adefovir) (Fig. 1), the prototype compound of another subclass of the acyclic nucleoside phosphonates, which is used for the treatment of hepatitis B virus infections [22] but has also shown to efficiently suppress HIV and HSV in cell culture [20], [21].

Human CD_4_^+^ T-lymphocyte CEM cell cultures were inoculated with HIV-1 and HIV-2 and exposed to adefovir, tenofovir, or PMEO-DAPym. All the compounds potently and comparably suppressed HIV replication with 50% effective concentration (EC_50_) values that ranged between 0.36 and 1.9 µg/ml (Table 1).

**Table 1:** Comparative antiviral activity of ANP derivatives in cell culture.

	EC_50_ a (µg/ml)			
	Adefovir	PMEO-DAPym	Tenofovir	Acyclovir
	(PMEA)		[(*R*)PMPA]	
CD4^+^ T-lymphocytes (CEM)				
HIV-1(III_B_)	0.96±0.24	0.9±0.4	0.36±0.24	>100
HIV-2(ROD)	1.9±1.1	0.66±0.19	0.43±0.41	>100
Human embryonic lung fibroblasts (HEL)				
HSV-1 (KOS)	4.7±1.8	2.6±1.2	105±20	0.049±0.027
HSV-2 (G)	6.3±2.6	4.4±2.4	122±51	0.053±0.033
HSV-1 (average of six clinical isolates)	6.2±1.0	2.0±0.5	125±12	0.05±0.01
HSV-2 (average of seven clinical isolates)	5.9±0.9	2.0±0.4	139±11	0.08±0.01
HSV-1 (average of six TK-deficient clinical isolates)	7.0±1.4	2.4±0.6	151±19	17±2
HSV-2 (average of seven TK-deficient clinical isolates)	7.5±1.5	2.4±0.4	121±15	21±2
Human monocyte/macrophages				
HSV-2 (G)	0.016±0.004	0.09±0.03	0.20±0.06	0.130±0.015

a 50% effective concentration (compound concentration required to inhibit virus-induced cytopathicity in cell culture by 50%).

**Figure 2:**
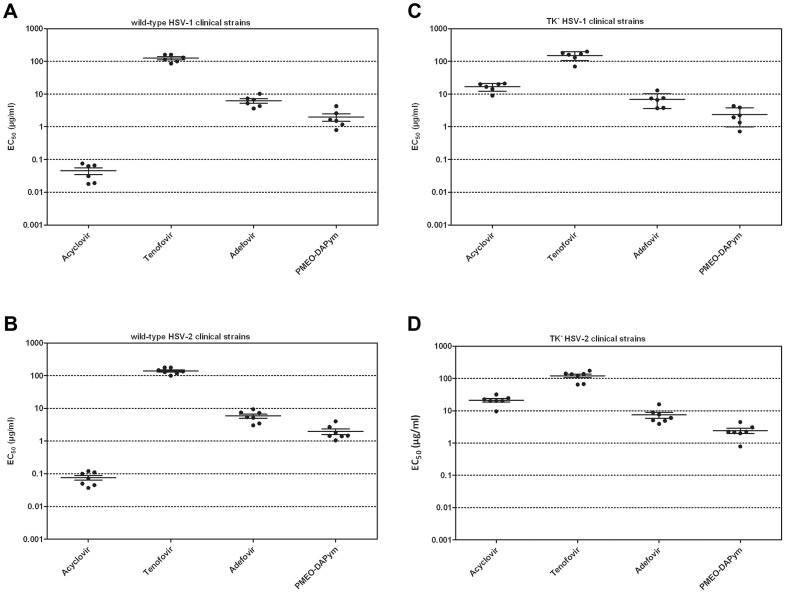
Antiherpetic activity of test compounds in cell culture. Confluent HEL cell cultures were exposed to 100 TCID_50_ of clinical virus isolates [wild-type HSV-1 (panel A), wild-type HSV-2 (panel B), TK^−^ HSV-1 (panel C) and TK^−^ HSV-2 (panel D)] in the presence of drugs at different concentrations and incubated for 3 days at 37°C. Then, the cytopathicity was determined microscopically and the EC_50_ values determined. ^a^EC_50_, 50% effective concentration or compound concentration required to reduce virus-induced cytopathicity (CPE) by 50%. Data shown are the means of at least two independent experiments. The HSV-1, HSV-2, HSV-1 TK^−^, and HSV-2 TK^−^ clinical isolates have been described in reference 19, including the nature of the mutations in the TK gene of the acyclovir-resistant virus strains.

The anti-HSV activities of PMEO-DAPym, adefovir, and tenofovir were evaluated in human embryonic lung HEL cell cultures infected with wild-type laboratory HSV-1 and HSV-2 strains. PMEO-DAPym and adefovir were 20- to 40-fold superior to tenofovir against HSV: the EC_50_ for adefovir and PMEO-DAPym ranged between 2.6 and 6.3 µg/ml compared with 105–122 µg/ml for tenofovir (Table 1). Comparable data were obtained when tested against thirteen clinical wild-type HSV-1 and HSV-2 isolates: on average the EC_50_ for PMEO-DAPym was ∼2 µg/ml, and for adefovir ∼6 µg/ml, while for tenofovir it was ∼130 µg/ml (Table 1, Fig. 2A,B). Also, both adefovir and PMEO-DAPym were 20- to 65-fold more effective than tenofovir against a broad variety of thirteen clinical mutant thymidine kinase-deficient (TK^−^) HSV-1 and HSV-2 strains. These clinical strains had been isolated from patients treated with anti-herpetic nucleoside analogues. The EC_50_'s of PMEO-DAPym (average EC_50_: 2.4 µg/ml) against these TK^−^ HSV isolates were lower (higher potency) than those of acyclovir (EC_50_: 17±1.9 µg/ml for HSV-1 (TK^−^) and 21±2.5 µg/ml for HSV-2 (TK^−^). (Fig. 2C,D). None of the compounds showed microscopical signs of cytotoxicity in the HEL cell cultures at the highest concentrations tested (i.e. 100 µg/ml).

### Antiviral activity in primary cell cultures

**Figure 3:**
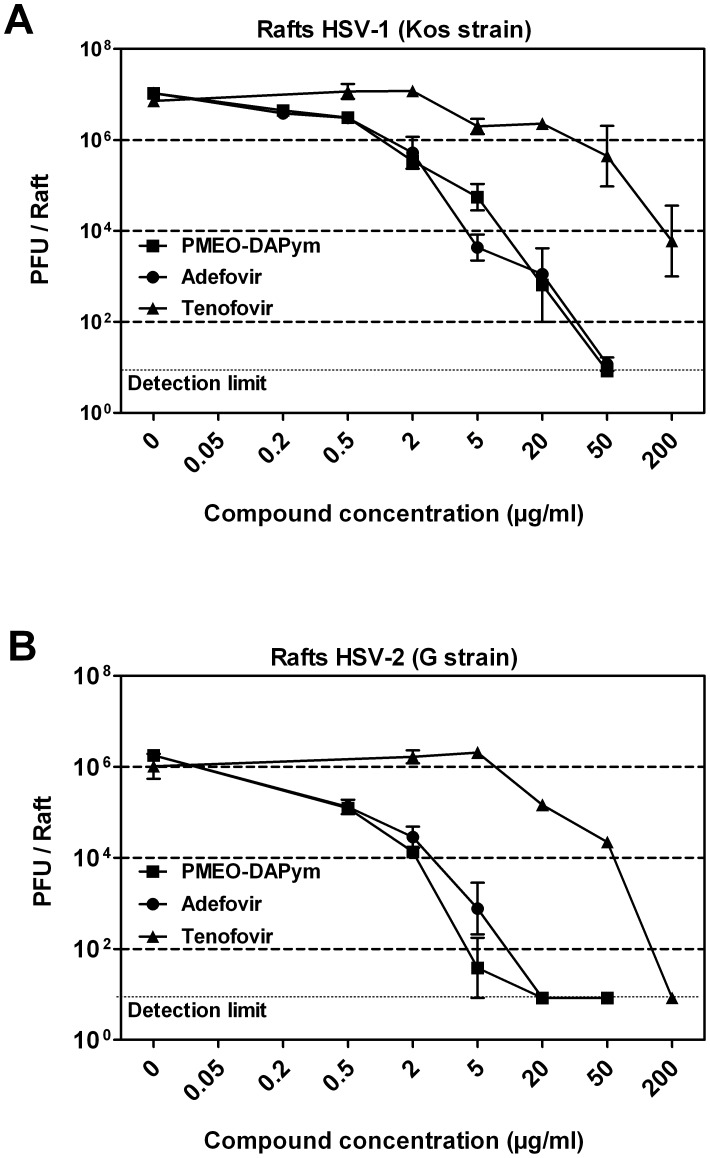
Inhibitory activities of tenofovir, adefovir, and PMEO-DAPym against laboratory HSV-1 and HSV-2 strains in organotypic epithelial raft cultures. Panels **A** (HSV-1) and **B** (HSV-2): Drugs at different concentrations were added to the raft cell cultures on the day of infection (10 days after initiation of differentiation). The drugs remained in the presence of the cells for 5 days until the rafts were frozen for determination of virus production with a plaque assay in HEL cell cultures. Error bars represent S.D.

**Figure 4:**
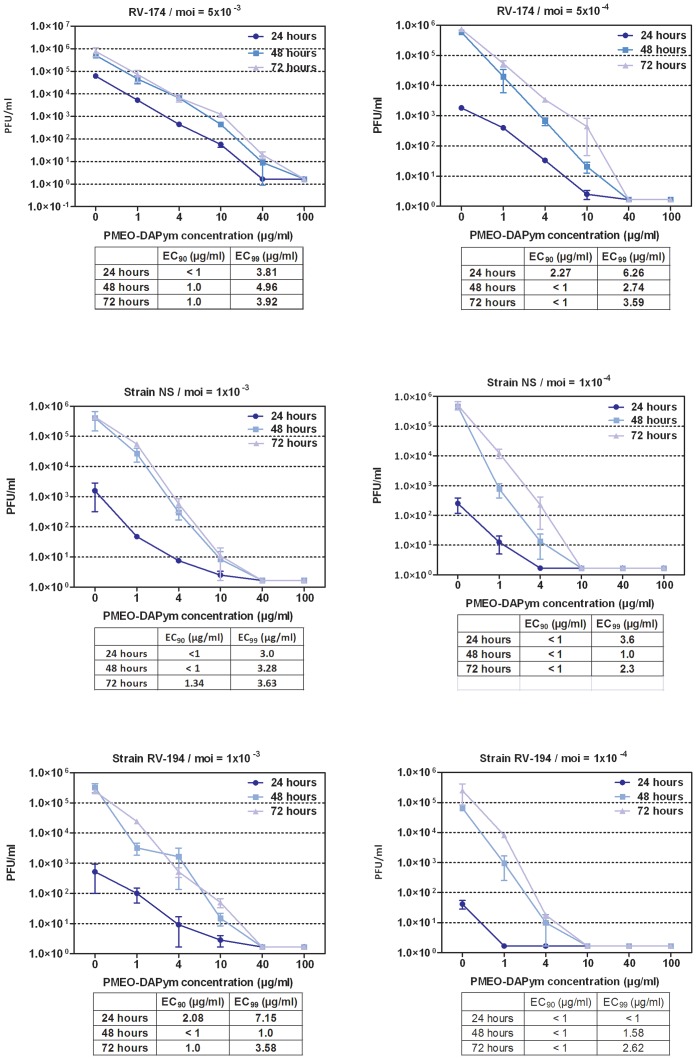
Inhibitory activity of PMEO-DAPym against clinical HSV isolates in HEL cell cultures. Different PMEO-DAPym concentrations were exposed to several wild-type clinical HSV-1 (RV-174) and HSV-2 (NS and RV-194) isolates in HEL cell cultures at different multiplicities of infection (m.o.i.; 10^−3^ (left graph) or 10^−4^ (right graph)). Virus yield was determined at 24, 48, and 72 h post infection, and EC_90_ and EC_99_ values were calculated from the graphical plots. Error bars represent S.D.

Primary human keratinocytes (PHK) are one of the main targets for HSV infection *in vivo*. Whereas 20 µg/ml tenofovir hardly affected HSV-1- and HSV-2-infected organotypic epithelial raft cultures of PHKs (<0.5 log_10_ reduction), a similar concentration of adefovir and PMEO-DAPym exhibited a striking 4 to 5 orders of magnitude reduction of herpesvirus replication (Fig. 3). In general, PMEO-DAPym inhibited the replication of the HSV-2 clinical isolates at EC_99_'s (viral load reduced by 2 orders of magnitude) between 1 and 7 µg/ml (Fig. 4), while EC_99_ inhibitory concentrations for tenofovir were in the range of 250 to 300 µg/ml [19]. Also, PMEO-DAPym or adefovir solely applied prior to virus infection resulted in a marked suppression of HSV replication. Acyclovir did not create such an anti-herpetic blockade upon preincubation (Table 2). The highest tested concentrations of PMEO-DAPym (i.e. 50 µg/ml) that fully suppressed HSV infection in the organotypic epithelial raft cultures of the PHKs had no effect on cell differentiation as ascertained by histological examination.

**Table 2:** Effect of pre-treatment of PHK (primary human keratinocyte) cell cultures with the drugs on herpesvirus infection.

Drug exposure time	HSV-1 (KOS strain)			HSV-2 (G strain)		
	Adefovir	PMEO-DAPym	Acyclovir	Adefovir	PMEO-DAPym	Acyclovir
Pretreatment −18 h to 0 h	8.2±1.6	7.0±0.6	>50	13.3±2.6	8.7±1.8	>50
Pretreatment −6 h to 0 h	23.3	29.6	>50	11.9	12.6	>50
Pretreatment −2.5 h to 0 h	30	31.6	>50	20.0	31.6	50
Pretreatment +2 h to 72 h	5.1±1.7	2.6±0.8	0.33±0.25	9.2±6.0	3.5±1.1	0.41±0.13

Confluent PHK cell cultures were pretreated with the drugs for different periods. Then, the drugs were carefully removed by several washing steps, after which the cultures were infected with HSV-1 or HSV-2. A control experiment in which the drugs were added to the virus-infected cultures for the entire incubation period before the read-out was also carried out (treatment condition: +2 h to 72 h).

a The 50% effective concentration (compound concentration required to inhibit HSV infection by 50%) as determined by scoring cytopathogenicity upon microscopical inspection.

Monocyte/macrophage (M/M) lineage cells can be infected by HSV-2 and HIV-1. M/M reside in genital herpes lesions and are thought to be reservoirs of HIV [6], [23]. In HSV-2-infected M/M, 2- to 10-µg/ml PMEO-DAPym, adefovir and tenofovir, virtually completely inhibited HSV-2 replication. Lower concentrations were dose-dependently inhibitory, with EC_50_ values ranging between 0.02 and 0.2 µg/ml (Table 1, Table 3). No effect on the viability of M/M cultures was observed for PMEO-DAPym at 100 µg/ml.

**Table 3:** Inhibitory activity of tenofovir, adefovir, and PMEO-DAPym against HSV-2 in primary M/M cultures as determined with the CPE reduction assay and the PFU reduction assay.

Treatment	Virus production by HSV2-infected macrophages	Inhibition of viral replication (% to control)	Cytopathicity (CPE) on macrophages
	6 days after infection		6 days after infection
	(CPE reduction) (TCID_50_/ml)		% CPE
**None**	1.4×10^4^		90
**PMEO-DAPym (µg/ml)**			
50	**No virus**	**100**	**-**
10	**No virus**	**100**	**0–5**
2	**2.26×10^2^**	**98–100**	**0–15**
0.4	**9.5×10^2^**	**93**	**35**
0.08	**7.5×10^3^**	**46**	**55**
0.016	**1.35×10^4^**	**3.5**	**80**
0.0032	**1.5×10^4^**	**-**	**85**
**Adefovir (µg/ml)**			
50	**No virus**	**100**	**-**
10	**No virus**	**100**	**-**
2	**No virus**	**100**	**0–5**
0.4	**8×10^2^**	**94**	**30**
0.08	**1.8×10^3^**	**87**	**40**
0.016	**7.2×10^3^**	**49**	**60**
0.0032	**1.6×10^4^**	**-**	**80**
**Tenofovir (µg/ml)**			
50	**No virus**	**100**	**-**
10	**No virus**	**100**	**10**
2	**3. 2×10^2^**	**98**	**30**
0.4	**1.9×10^3^**	**86**	**40**
0.08	**9×10^3^**	**36**	**60**
0.016	**1.3×10^4^**	**7**	**75**
0.0032	**1.2×10^4^**	**14**	**85**
**Acyclovir (µg/ml)**			
0.08	**No virus**	**100**	**-**
0.016	**No virus**	**100**	**-**
0.003	**No virus**	**100**	**-**
0.00064	**3.5×10^2^**	**97**	**10**
0.000128	**7.2×10^3^**	**49**	**40**
0.000025	**1.3×10^4^**	**7.2**	**75**

Virus production was evaluated on day 6 post infection. Infectious HSV-2 (G) was quantified in the supernatants of drug-treated virus-infected cell cultures by titration in Vero cell cultures using the CPE reduction assay (TCID_50_/ml). The inhibition of viral replication was expressed in percentages and calculated with respect to the virus production in infected untreated macrophages. The inhibition of cytopathicity by the drugs was evaluated from microscopic inspection and was in agreement with the dose-dependent reduction of virus production. The data shown are from one representative experiment that was independently repeated three times.

### Antiviral activity in human ex vivo lymphoid tissue

**Figure 5:**
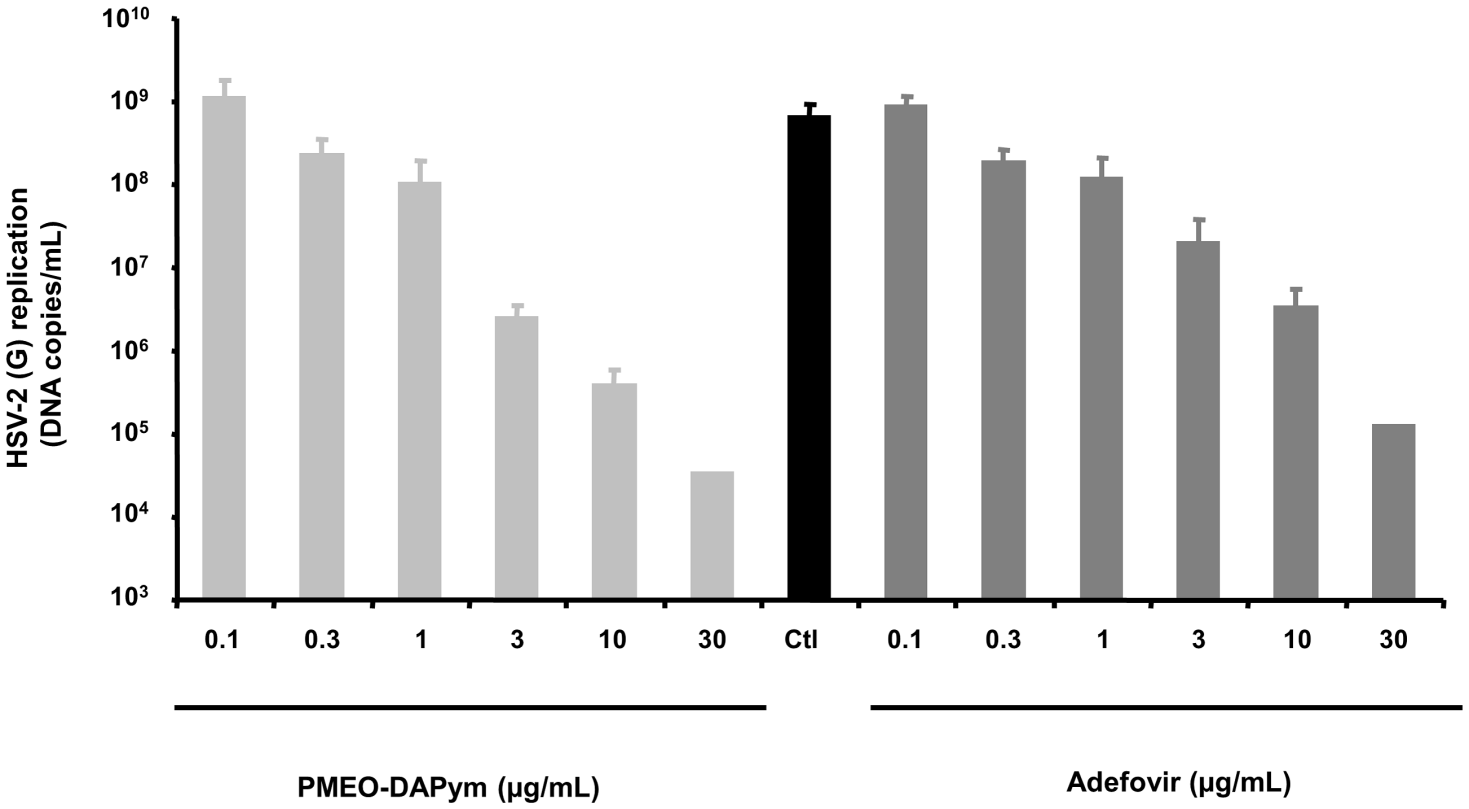
Suppression of HSV-2 in infected human *ex vivo* tonsillar tissue by adefovir and PMEO-DAPym. Blocks of human tonsillar tissue were inoculated *ex vivo* with HSV-2 (G) and treated or not with adefovir or PMEO-DAPym. We monitored HSV-2 (G) replication by measuring viral DNA in culture media at different times throughout the culture period. Presented are means ± SEM of cumulative HSV-2 (G) replication in tissues from two to six donors. For each donor, data represent pooled viral release from 27 tissue blocks.

**Figure 6:**
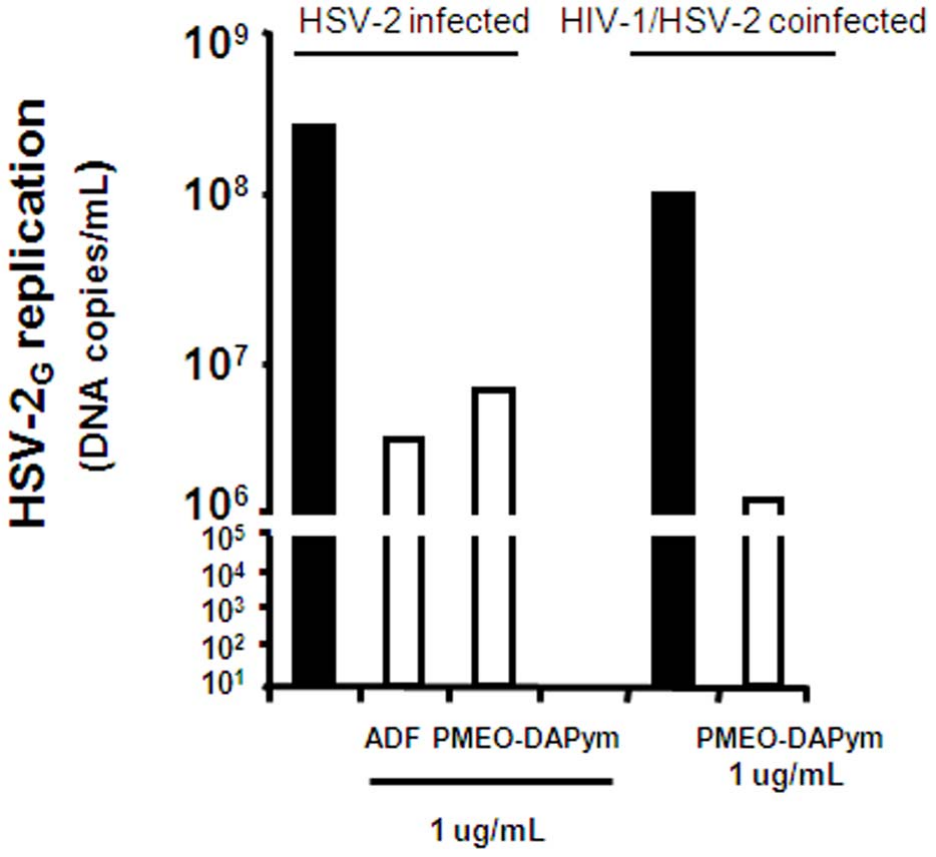
Suppression of HSV-2 in human cervico-vaginal tissues by adefovir and PMEO-DAPym. Blocks of human cervico-vaginal tissues were inoculated *ex vivo* with HSV-2 (G) or co-infected with HIV-1 and HSV-2 and treated or not with adefovir (1 µg/ml) or PMEO-DAPym (1 µg/ml). We monitored HSV-2 (G) replication by measuring viral DNA accumulated in culture media at different times throughout the culture period. Presented is cumulative HSV-2 (G) replication, with data representing pooled viral release from 16 tissue blocks.

**Figure 7:**
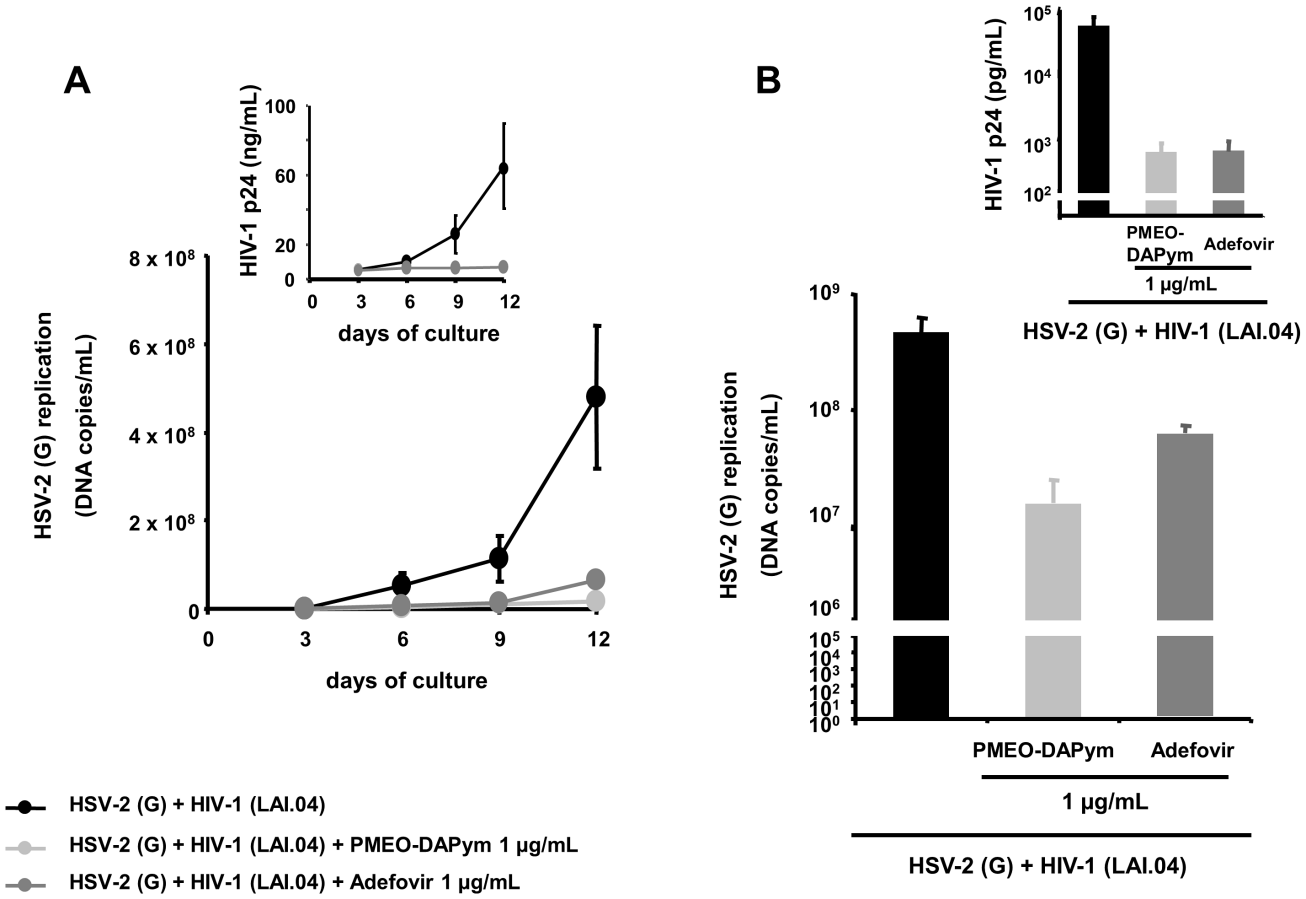
Suppression of HSV-2 in single-infected and HIV-1 co-infected human *ex vivo* tonsillar tissue by adefovir and PMEO-DAPym. Panels A and B: Blocks of human tonsillar tissue from five different donors were co-inoculated *ex vivo* with HSV-2 (G) and HIV-1 (LAI) and treated or not with adefovir or PMEO-DAPym (1 µg/ml). For tissue from each donor, we monitored replication of both viruses by evaluating their accumulation in the culture media bathing 27 tissue blocks at different times throughout the culture. We evaluated HIV-1 replication by measuring p24_gag_ and HSV-2 (G) replication by measuring HSV-2 viral DNA. Panel **A**: Presented are kinetics of HSV-2 (G) and HIV-1 (LAI) replication (insert). Each point represents the mean ± SEM of viral replication in tissues from five donors. Panel **B**: Presented are means ± SEM of cumulative HSV-2 (G) and HIV-1 (LAI) replication (insert).

Human lymphoid tissue explants that retain tissue cytoarchitecture and many of its physiological functions represent one of the most adequate laboratory models for studying viral pathogenesis [24]. Adefovir and PMEO-DAPym inhibited HSV-2 replication in this tonsilar system at an EC_99_ (i.e., viral load reduced by 2 orders of magnitude) of 5 µg/ml and 2 µg/ml, respectively, as determined with an RT-PCR assay (Fig. 5). HSV-2 inhibition by 4 to 5 orders of magnitude was observed for both drugs at 30 µg/ml. In contrast, tenofovir comparably inhibited HSV-2 replication at much higher concentrations (240 µg/ml) [19]. Also in cervico-vaginal explants, both adefovir and PMEO-DAPym at 1 µg/ml markedly inhibited HSV-2 infection (Fig. 6). To mimic more closely the *in vivo* situation, we coinfected human *ex vivo* tissue with HIV-1 and HSV-2. In both lymphoid and cervico-vaginal tissues, the replication of both viruses was efficiently suppressed by 1 µg/ml adefovir or PMEO-DAPym as observed from the production of HSV-2 DNA and HIV-1 p24 Ag in the culture supernatants (Fig. 6. and 7A,B). In coinfected lymphoid tissues, the HSV-2 load was reduced by 1 to 1.5 orders of magnitude by adefovir and PMEO-DAPym, while HIV-1 was partially inhibited by either of these drugs by 2 orders of magnitude (Fig. 7B). Although extremely potent (99.34% inhibition at 3 µg/mL), PMEO-DAPym was still less efficient to inhibit HSV-2 in human lymphoid tissue compared to ACV (Table 4).

**Table 4:** Inhibitory activity of PMEO-DAPym and acyclovir against HSV-2 in human lymphoid tissue *ex vivo*.

Treatment	HSV-2 DNA copies	Inhibition of viral replication (% to control)
None	1.22 e+08	
PMEO-DAPym (µg/mL)		
3	8.08 e+5	99.34%
0.3	5.76 e+7	52.79%
ACV (µg/mL)		
3	2.83 e+4	99.98%
0.3	1.69 e+5	99.86%

### In vivo antiherpetic activity

**Figure 8:**
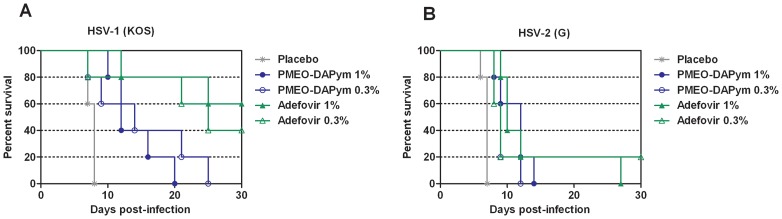
Effects of adefovir and PMEO-DAPym on mortality in mice inoculated with HSV-1 or HSV-2. Groups of five nu/nu mice were inoculated with HSV-1 (panel A) or HSV-2 (panel B) on the lumbosacral area. Each cohort was then subjected to topical treatment twice daily for 5 consecutive days, starting on the day of viral infection. The placebo groups received a similar treatment with the test formulation without drug. Mortality was recorded over a period of 30 days. Animals were euthanized when more than 30% loss in body weight or development of paralysis occurred. We estimated survival rates according to the Kaplan-Meier method and compared them using the log-rank test (Mantel-Cox using GraphPad Prism). Several curves proved statistically significant (*p*<0.01) in comparison of each treatment with placebo.

Athymic nude mice lumbosacrally scarificated with HSV-1 or HSV-2 were used for testing adefovir and PMEO-DAPym (Fig. 8). Placebo-treated mice developed lesions at the lumbosacral area leading to paralysis of the hind legs, and animal death occurred within 7 to 8 days. Treatment with either one of the drugs significantly delayed virus-related morbidity and prolonged the survival of the mice (*p*<0.05) (Fig. 8A,B). Although adefovir better suppressed HSV infection than PMEO-DAPym in the virus-infected mice, both drugs were superior to tenofovir in their antiviral efficacy [19].

Thus, PMEO-DAPym and to a certain extent adefovir demonstrated a unique potency in efficiently suppressing HIV and HSV in *in vitro*, *ex vivo*, and *in vivo* experimental systems.

### Inhibitory activity against virus-encoded DNA polymerases

The pharmacologically active diphosphorylated metabolites of adefovir and PMEO-DAPym efficiently inhibited HIV RT– and HSV DNA polymerase–catalysed [^3^H]dATP incorporation at 50% inhibitory concentrations (IC_50_) of 0.19 and 0.43 µg/ml for HIV-1 RT and 0.43 and 0.52 µg/ml for HSV DNA polymerase, respectively (Tables 5 & 6). IC_50_'s for DNA polymerization by incorporation of other [^3^H]dNTPs by HSV DNA polymerase were, respectively, 1.5–2.1 and 3.1–5.2 µg/ml for adefovir-diphosphate and PMEO-DAPym-diphosphate, whereas in this system the IC_50_ for tenofovir-diphosphate was markedly higher (8.9–32 µg/ml) (Table 6). Thus, efficient suppression of both HIV RT and HSV DNA polymerase by PMEO-DAPym explains its concomitant anti-HIV-1 and anti-herpetic activity.

**Table 5:** Inhibitory activity of the diphosphate metabolites of adefovir, tenofovir, and PMEO-DAPym against HIV-1 RT.

Compound	IC_50_ a (µg/ml)
Adefovir-pp	0.40±0.30
PMEO-DAPym-pp	0.19±0.21
Tenofovir-pp	0.43±0.21

a 50% Inhibitory concentration (drug metabolite concentration required to inhibit HIV-1 RT-catalysed DNA polymerization by 50%), using dATP (3.2 µM) as the competing substrate and poly rU.dA as the primer/template.

**Table 6:** Inhibitory activity of the diphosphate metabolites of adefovir, tenofovir, and PMEO-DAPym against HSV DNA.

Compound	IC_50_ a (µg/ml)			
	dATP	dCTP	dGTP	dTTP
Adefovir-pp	0.43±0.14	1.7±0.1	1.5±1.0	2.1±1.1
PMEO-DAPym-pp	0.52±0.03	5.2±4.8	3.1±1.0	5.2±0.3
Tenofovir-pp	0.48±0.04	32±2.2	8.9±6.3	11±4.8

a Four separate experiments were carried out in which three non-radiolabeled dNTPs were present at 100 µM and the fourth dNTP was radiolabeled and present at 1 to 3.2 µM depending on the nature of the [^3^H]dNTP. The 50% inhibitory concentration was defined as the drug metabolite concentration required to inhibit HSV DNA polymerase-catalysed DNA synthesis by 50%, using different (radiolabeled) dNTPs as direct competing substrates. As the primer/template, gapped calf thymus DNA was used.

### Immunomodulatory activity of PMEO-DAPym

**Figure 9:**
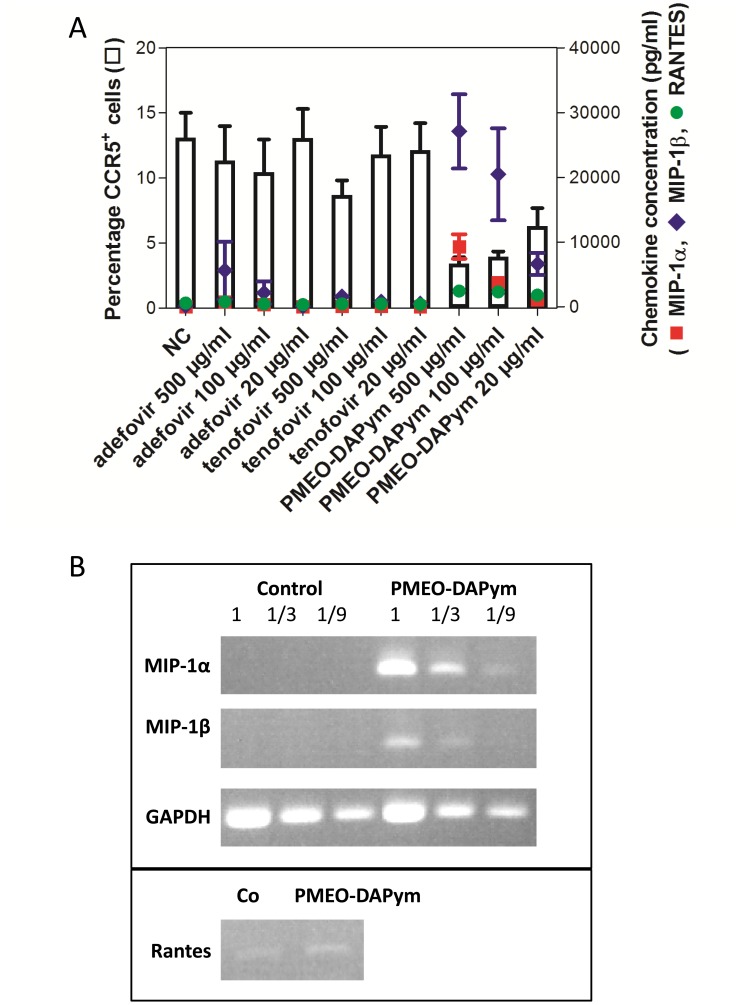
Expression of CC-chemokines, CCR5 and chemokine mRNA expression in PBMC cultures after drug treatment. Panel **A**: Production of CC-chemokines by PBMC and the effects of adefovir, tenofovir, and PMEO-DAPym on the expression of the CCR5 receptor. Freshly isolated PBMCs from four healthy donors were incubated for 24 h with medium only (NC) or with adefovir, tenofovir, or PMEO-DAPym. We collected the supernatants and measured the concentrations of MIP-1α (▪), MIP-1β (▴), and RANTES (•) using a Bioplex system (Bio-Rad, Hercules, CA). Shown are means ± SEM. The cells were also collected, and the expression of the CCR5 receptor (□) was measured with flow cytometry using the PE-labeled CCR5 (clone 2D7) mAb; it is shown as percentage of control (± SEM) from four independent representative experiments. Panel **B**: Chemokine mRNA expression upon PMEO-DAPym exposure to PBMC. RT-PCR analysis of MIP-1α, MIP-1β, RANTES, and GAPDH (control) expression in PBMC treated with 100-µg/ml PMEO-DAPym for 4 h. Similar results were obtained with PBMC from three different donors. Results from one representative donor are shown.

**Figure 10:**
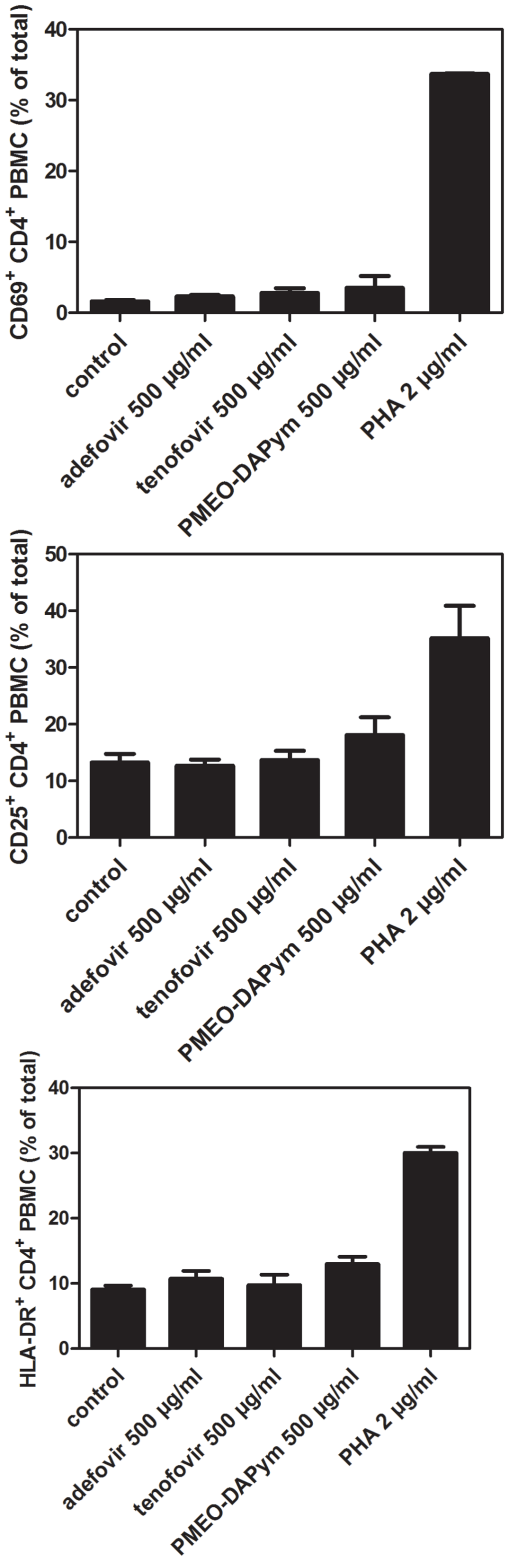
Expression of activation markers on PBMCs treated with adefovir, tenofovir, or PMEO-DAPym. PBMCs were incubated with adefovir, tenofovir, or PMEO-DAPym for 3 days. The mitogenic lectin PHA (at 2 µg/ml) was included as a positive control. We measured cell surface expression of CD4 and the activation markers CD69 (top panel), CD25 (middle panel), and HLA-DR (bottom panel) using flow cytometry with the fluorescein isothiocyanate-labeled CD4-specific mAb (clone SK3) and the phycoerythrin-labeled CD69, CD25, HLA-DR-specific mAbs. Shown are means ± SEM from two independent experiments.

Although the suppression of HIV-1 RT and HSV DNA polymerase seems to be sufficient to explain the dual-targeted anti-viral activity of the tested compounds, we discovered an additional immune-modulatory mechanism for PMEO-DAPym that may further contribute to the anti-HIV-1 activity of this drug: PMEO-DAPym strikingly induces the release of anti-HIV-1 CC chemokines in peripheral blood mononuclear cells (PBMCs) in a dose-dependent manner (Fig. 9A). At a concentration as low as 20 µg/ml, PMEO-DAPym induced 7.5 ng/ml MIP-1β in the PBMC cultures at 24 h after drug exposure, while at 100 and 500 µg/ml, it induced the release of 20- and 27-ng/ml MIP-1β, respectively. These PMEO-DAPym concentrations did not affect PBMC viability as measured by the trypan blue dye exclusion method and flow cytometric analysis. PMEO-DAPym also dose-dependently induced secretion of MIP-1α and RANTES, although at concentrations inferior to those of MIP-1β. PMEO-DAPym at 100 µg/ml caused a >9-fold increase in MIP-1β and MIP-1α mRNA expression (Fig. 9B), pointing to the necessity of triggering new mRNA synthesis to enable the upregulation of these CC-chemokines. Neither adefovir nor tenofovir induced notable production of CC-chemokines even at 500 µg/ml. Note that the secretion of the CC-chemokines was not the result of the stimulation of activation markers, since none of the cell activation markers included in our studies (i.e. HLA-DR, CD25 and CD69) (Fig. 10), neither CD3, CD28, CD38, CD54, CD71, CD80 and CD86 (data not shown) were upregulated by this drug after 72 h exposure to PBMC.

**Figure 11:**
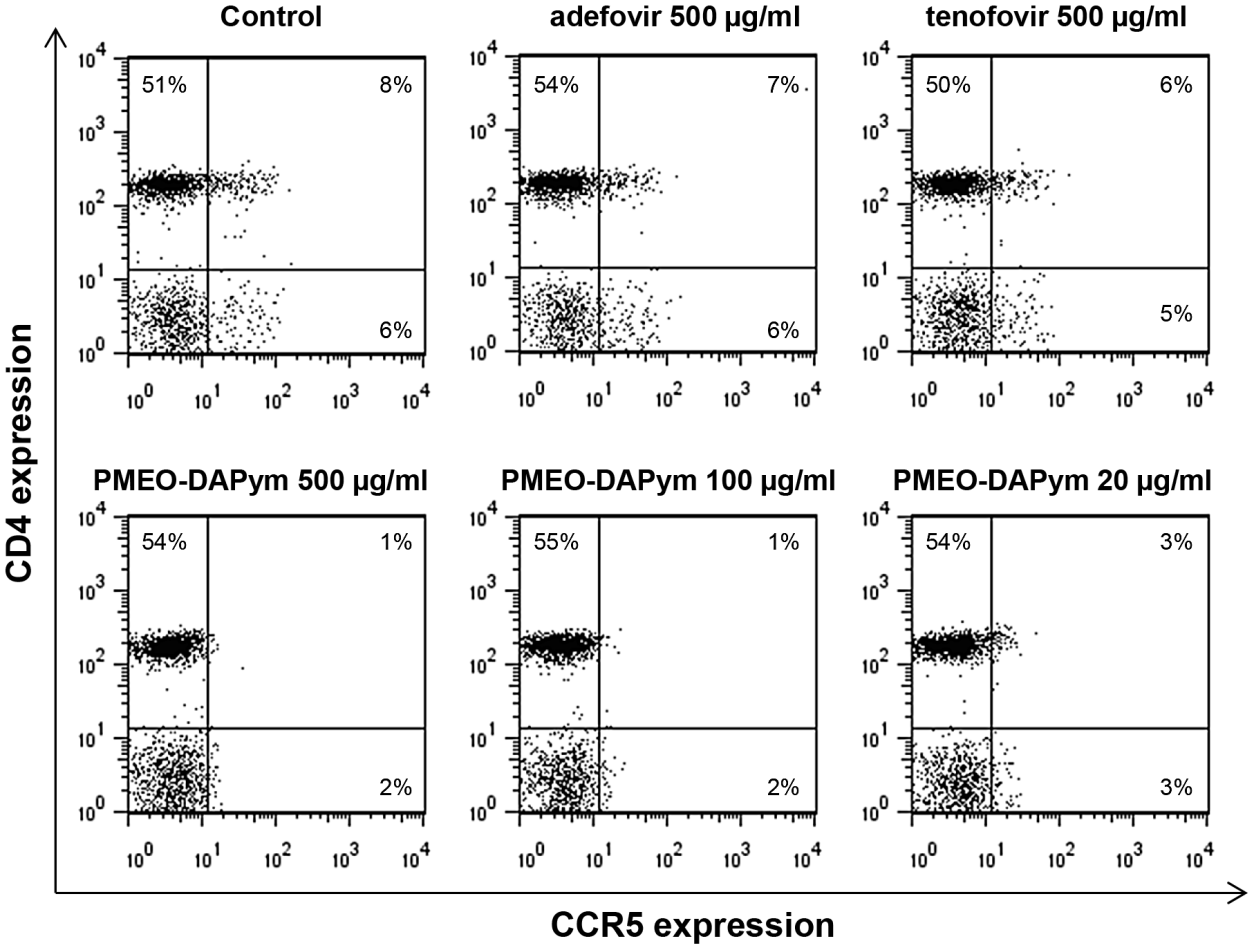
Expression of the HIV-1 coreceptor CCR5 on PBMCs after treatment with adefovir, tenofovir, or PMEO-DAPym. We treated PBMC with the drugs for 24 h and collected and analyzed the cells using flow cytometry. Cell surface expression of CD4 and the chemokine receptor CCR5 were measured with the fluorescein isothiocyanate-labeled CD4 specific mAb (clone SK3) and the phycoerythrin-labeled CCR5 (clone 2D7). The percentages of positive cells in each quadrant of the dot plots are given. The data shown are from one representative experiment that was independently repeated at least four times.

**Figure 12:**
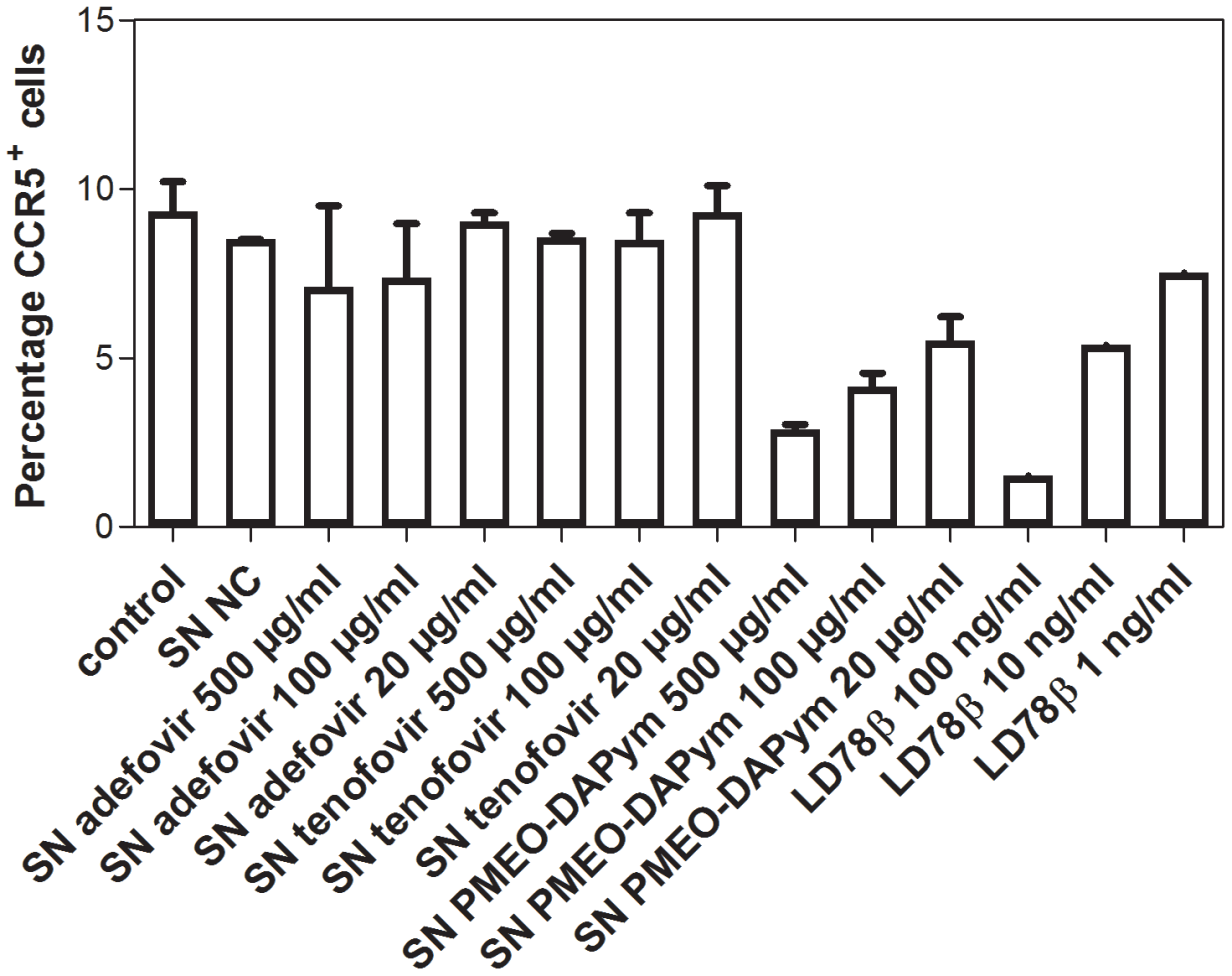
Effects of the supernatants collected after incubation of PBMCs with adefovir, tenofovir, and PMEO-DAPym on the expression of CCR5. PBMCs were treated with medium alone or with various concentrations of adefovir, tenofovir, or PMEO-DAPym, and the supernatants were collected after 24 h. Then, freshly isolated PBMCs were incubated at 37°C for 1 h with these various supernatants, with medium alone, or with LD78β (control) at 100 ng/ml, 10 ng/ml, and 1 ng/ml. We measured the expression of the CCR5 receptor using flow cytometry with the phycoerythrin-labeled CCR5 (clone 2D7) mAb: shown are the percentages (means ± SEM for two independent experiments) of CCR5+ cells.

Importantly, PMEO-DAPym-induced production of CC chemokines was associated with a decrease of CCR5 expression on the cell surface by ≥75% at 500 and 100 µg/ml, and by 60% at 20 µg/ml) (Fig. 9A,11). Neither adefovir nor tenofovir induced a marked CCR5 decrease. The observed downregulation of CCR5 by PMEO-DAPym proved to be induced by the released CC-chemokines themselves, as the conditioned medium from PMEO-DAPym-exposed PBMC markedly and dose-dependently decreased CCR5 expression in freshly exposed PBMCs within 1 h of exposure time. This effect was not due to residual amounts of PMEO-DAPym in the conditioned medium, since exposure of PBMC to this drug for 1 h was not sufficient to downregulate CCR5 (data not shown). Exogenous recombinant MIP-1 α (isoform) LD78β showed an effect on the PBMC cultures similar to that of PMEO-DAPym. In contrast, conditioned medium from either adefovir- or tenofovir-exposed PBMC was unable to decrease CCR5 expression in freshly exposed PBMCs (Fig. 12). No downregulation of CXCR4 or CXCR3 was observed (data not shown), pointing to an important degree of (co)receptor selectivity by PMEO-DAPym. It should also be mentioned that exposure of PBMC to longer time periods than 24 h (i.e. 72 h) still showed a significant drop of CCR5 expression in the PBMC cultures. Indeed, CCR5 expression after 72 h was 10%, 38% and 60% of control after addition of 500, 100 and 20 µg/ml PMEO-DAPym. Thus, the target cells do not compensate for the drug-induced CCR5 drop, and PMEO-DAPym differs in this respect with some co-receptor antagonists for which CCR5 down-regulation recovered after longer time periods. Beside β-chemokine induction, PMEO-DAPym also triggered the expression of several other chemo/cytokines and growth factors in a dose-dependent manner (Fig. S1). These stimulatory effects were less pronounced in PHA-exposed PBMC (Fig. S2).

## Discussion

We demonstrated the potent anti-HIV and -HSV activity of PMEO-DAPym in a diverse set of clinically relevant *in vitro*, *ex vivo*, and *in vivo* systems including (i) CD4^+^ T-lymphocyte (CEM) cell cultures, (ii) embryonic lung (HEL) cell cultures, (iii) organotypic epithelial raft cultures of primary human keratinocytes (PHKs), (iv) primary human monocyte/macrophage (M/M) cell cultures, (v) human *ex vivo* lymphoid tissue, and (vi) athymic nude mice. In all assay systems the drugs were administered prior to, or at the time of, virus infection which is relevant from a microbicidal application viewpoint, for which the drugs are preferentially already present at the time of infection. However, it might well be possible that these drugs are also effective when exposed shortly after virus infection given the DNA polymerase/HIV reverse transcriptase as being one of the intracellular targets of PMEO-DAPym. In addition to our findings that PMEO-DAPym can efficiently suppress a wide variety of HSV clinical isolates including acyclovir-resistant virus strains, it has previously been demonstrated that PMEO-DAPym suppresses a wide variety of HIV-1 clinical strains belonging to different HIV-1 clades (i.e. A, B, C, A/E, G) [25]. This study also revealed that PMEO-DAPym showed a somewhat more favorable cross-resistance profile to various mutant HIV-1 isolates than adefovir and tenofovir. Likewise, we could demonstrate that the antivirally active PMEO-DAPym diphosphate metabolite potently inhibited mutant HIV-1 RT enzymes that harbor the tenofovir-characteristic K65R and K70R mutations (IC_50_ in the submicromolar range) (data not shown) explaining the efficient suppression of a variety of (mutant) clinical virus isolates. Thus, PMEO-DAPym may have a rather high genetic barrier suppressing a wide variety of clinical HSV and HIV-1 clade isolates, including clinically relevant mutant viruses.

We investigated the underlying basis of the antiviral activity of PMEO-DAPym and found several different mechanisms that together explain the unique dual antiviral activity of this new compound: PMEO-DAPym efficiently suppressed HIV reverse transcriptase (RT)– and HSV-1 DNA polymerase–catalysed viral replication. Moreover, it also induced secretion of beta-chemokines, in particular MIP-1β, MIP-1α, and RANTES, whose upregulation is associated with a pronounced downmodulation of the HIV-1 coreceptor CCR5, resulting in inhibition of HIV entry.

PMEO-DAPym did not show benefit over adefovir in HSV-infected mice (Fig. 8). These experiments have been performed in immunodeficient athymic nude mice. However, PMEO-DAPym was approximately equally effective as adefovir in human cervico-vaginal tissues *ex vivo* (Fig. 6) and slightly superior to adefovir in human tonsillar tissue *ex vivo* (Fig. 7). The main conclusion of the antiviral data in the *ex vivo* and *in* vivo models is that PMEO-DAPym is superior to tenofovir in its antiherpetic activity. Since it is approximately equal to tenofovir for its anti-HIV activity, PMEO-DAPym can therefore be considered as an efficient dual-targeted antiviral.

Thus, this study revealed an additional and surprising property of PMEO-DAPym that is of clinical importance especially in view of its potential microbicide application. Indeed, the immunomodulatory/immunostimulatory properties of ANPs such as PMEO-DAPym may become highly relevant in case of microbicide drug development. A growing body of evidence in fact suggests that dual antiviral mechanisms should be required for effective mucosal protection [26], including stimulation of innate antiviral factors such as β-chemokines that down-regulate the HIV CCR5 co-receptor [27], [28]. It was shown that RANTES, MIP-1α and MIP-1β are significantly associated with protection against rectal mucosal SIV infection [29]. RANTES derivatives are currently considered for microbicide formulation. Dudley et al. [30] could show that chemically modified RANTES, PSC-RANTES, binds to CCR5, inhibits HIV-1 entry and dose-dependently protects rhesus macaques from SHIV infection as a vaginal microbicide. Ahmed et al. [31] demonstrated that spontaneous production of RANTES and IFN-γ correlated with protection against SIV_sm_ challenge of Cynomolgus macaques.

Beside the pronounced stimulatory effect of PMEO-DAPym on MIP-1α and MIP-1β, and to a lesser extent RANTES, it also stimulates the production of a variety of other interleukins and several growth factors. Its stimulatory effect on the mitogenic IL-2 and IL-4 chemokines, as well as IL-7 that inhibits apoptosis of HIV-infected cells and stimulates T-cell proliferation [32], is very minor. Also, PMEO-DAPym did not affect IP-10 levels. This chemokine is a potent chemotactic factor for lymphocytes and monocytes [33] and thus, the drug is not expected to attract these cells to the genital sub-mucosa through IP-10 induction. Instead, the induction of proinflammatory interleukins such as IL-6 and IL-8 is more pronounced in cells treated with PMEO-DAPym. Also production of G-CSF and IL-1β was markedly enhanced in the drug-exposed PBMC cultures. In contrast with the activation of P2Y2 nucleotide receptors with UTP resulting in an intracellular Ca^++^ response to activation of CXCR2 in HEK cells [34], the broad cellular chemokine response triggered by PMEO-DAPym does not induce Ca^++^ release in drug-exposed PBMC (data not shown). However, the relevance of the stimulatory effect on these interleukins in PBMC by PMEO-DAPym suggesting potential side effects of the drug is currently still unclear. In fact, PRO-2000, a polyanionic drug that has been exposed to healthy individuals for a prolonged time period as a potential microbicide drug also markedly stimulated IL-1ra, IL-6 and IL-8, G-CSF and GM-CSF [35] in PBMC, but was never found to display any signs of toxicity or HIV-promoting transmission in the drug-treated individuals [36].

The fact that (i) the CC chemokines RANTES, MIP-1α and MIP-1β act as potent natural inhibitors of HIV-1 [37], [38], (ii) such chemokines are indeed protective to SIV challenge in monkeys by acting as virus entry inhibitors [29], [39], (iii) ANPs, in particular PMEO-DAPym, may behave as natural chemokine production enhancers in PBMC and M/M in addition to their viral reverse transcriptase/DNA polymerase inhibition, (iv) the increased β-chemokine production found in PMEO-DAPym-exposed PBMC cultures coincided with CCR5 co-receptor down-regulation and (v) the supernatants of these drug-exposed cell cultures were able to induce an antiviral refractory state when administered to new non-treated cell cultures, point to an additional and important antiviral (i.e. HIV) potential of PMEO-DAPym through its immunomodulatory properties. In our experiments, it was shown that 500 and 100 µg/ml PMEO-DAPym down-modulated CCR5 by ≥75%. This extent of CCR5 down-regulation might be sufficient to affect virus infection, since studies with anti-CCR5 antibodies have demonstrated that their exposure to PBMC can drop the mean surface expression of CCR5 from CD4^+^ T-lymphocytes or U87-CCR5 cell cultures to 19–37% of control. In such cases, the antibodies could efficiently neutralize R5 virus infection [40]. Although the exact molecular mechanism of action of PMEO-DAPym is yet to be revealed, it is clear that its action on CCR5 down-regulation by stimulation of β-chemokine mRNA and subsequent enhanced β-chemokine induction is different from the previously reported down-modulation of CCR5 (and concomitant anti-HIV activity) by rapamycin. The latter drug, that disrupts IL-2 receptor signaling, interferes with CCR5 expression at the transcriptional level, and the reduced expression of CCR5 on PBMCs is then associated with increased extracellular levels of MIP-1α and MIP-1β [41]. It should be kept in mind that intravaginal 1% gel applications (as used in the CAPRISA 004 trial) afford high local drug concentrations at the cervicovaginal tissue that easily exceed those used in our study [42], [43]. Therefore, it is expected that such locally high drug concentrations may efficiently induce β-chemokine production that, in turn, will be able to down-regulate CCR5 expression, creating a local transient antiviral state in addition to efficient inhibition of the viral DNA polymerases of HIV and HSV. It should be emphasized that our study has not addressed other key aspects that are important for eventual clinical efficacy of a drug, such as the optimal drug concentration that can be reached, the drug formulation, its pharmacokinetics and pharmacodynamics, and potential drug-resistance development. These aspects are somewhat complicated given the fact that PMEO-DAPym targets two different viruses and regarding the anti-HIV activity, PMEO-DAPym is directed against two different events in the viral infection/replication cycle.

The antiherpetic activity of PMEO-DAPym is inferior to that of acyclovir (Table 1, 3 and 4). However, in human tissues *ex vivo* at the concentration of 3 µg/mL both drugs suppressed HSV-2 by more than 99%. Future modifications of the drug may bring it to an anti-HSV activity even closer to that of acyclovir, the highly efficient anti-HSV compound and the current drug of choice to treat HSV-2 infection. The key accomplishments in designing PMEO-DAPym is its anti-HIV activity comparable to that of tenofovir combined with a marked (∼30- to 70-fold) superior activity against HSV compared with tenofovir. Since tenofovir proved already effective to prevent HSV infection by 51% in the CAPRISA trial, it is expected that PMEO-DAPym will perform markedly better than tenofovir against HSV infection/transmission based on our *in vitro*, *ex vivo* and *in vivo* data. Although it would be preferable to develop a drug that concomitantly has potent anti-HIV and anti-HSV activity as the best in their class, it might be sufficient to have moderate activity against one of these viruses in case of microbicides given the topical administration modality that allows to reach high local (and therefore very effective) drug concentrations, as already shown for tenofovir in the CAPRISA trial [16], [17]. Also an important advantage of PMEO-DAPym is its suppression of CCR5-tropic HIV, the type of HIV-1 that is predominantly transmitted.

In conclusion, we defined a distinct new subclass of ANPs, structurally and functionally different from tenofovir and adefovir, that has significant advantage over the commonly used drugs. The prototype drug, PMEO-DAPym, efficiently suppresses HSV DNA polymerase and also retains the ability of tenofovir and adefovir to markedly suppress HIV-1 RT. In addition, and unlike adefovir and tenofovir, the drug also induces anti-HIV CC-chemokines, which downmodulate the CCR5 coreceptor. Thus, PMEO-DAPym combines various anti-HIV and anti-HSV activities in one molecule and concomitantly targets HIV entry and viral polymerase-catalysed HIV/HSV replication. The efficiency of this new class of antivirals (even at concentrations much lower than the ones achievable in vaginal application) [43] makes it a promising new-generation multitargeted and multifunctional antiviral for dual-viral (HSV/HIV) infection therapy and HIV transmission prevention.

## Methods

### Ethics statement

All animal work was approved by the Katholieke Universiteit Leuven Ethics Committee for Animal Care and Use (Permit number: P097-2010). All animal guidelines and policies were in accordance with the Belgian Royal Decree of 14 November 1993 concerning the protection of laboratory animals and the European Directive 86-609-EEC for the protection of vertebrate animals used for experimental and other scientific purposes.

Virus isolates were obtained as part of a translational research program (www.regavir.org) granted by the Belgian Ministry of Health as part of the National Cancer Plan for the diagnosis of drug resistance in herpesviruses. All viruses were obtained and used as approved by the Belgian IRB equivalent (Departement Leefmilieu, Natuur en Energie, protocol SBB 219 2011/0011, and the Biosafety Committee KU Leuven).

### Cells

Human embryonic lung HEL-299 fibroblasts were obtained from ATCC. Primary human keratinocytes (PHKs) were isolated from neonatal foreskins and cultured as previously described [44]. The TZM-Bl cells [45] were kindly provided by Dr. G. Van Ham (ITG, Antwerp, Belgium).

Buffy coat preparations from healthy donors were obtained from the Blood Transfusion Center in Leuven, Belgium. PBMC were isolated by density gradient centrifugation over Lymphoprep (d = 1.077 g/ml) (Nycomed, Oslo, Norway) and cultured in cell culture medium (RPMI 1640) containing 10% FCS and 2 mM L-glutamine. The healthy donors were anonymous.

### Viruses

The laboratory HSV-1 strain KOS and the HSV-2 strain G were used as reference herpesviruses. Several clinical isolates of wild-type HSV-1 [RV-6, RV-132, RV-134, C559143, RV-174, RV-175], thymidine kinase-deficient (TK^−^) HSV-1 [RV-28, RV-36, RV-117, 328058, RV-179, RV-294], wild-type HSV-2 [RV-24, RV-124, RV-194, NA, PB, NS, HSV-47], and HSV-2 TK^−^ [RV-101, RV-129, BA 19026589, LU C557672, HSV-44, RV-184, RV-185] from virus-infected individuals in Belgium were used. Viral TK sequences were deposited in Genbank (GenBank accession JN415116-JN415119 for HSV-1 mutants and JN415120–JN415126 for HSV-2 mutants). HIV-1 strains III_B_ and Ba-L were provided by Drs. R.C. Gallo and M. Popovic (at that time at the National Institutes of Health, Bethesda, MD) and HIV-2 (ROD) was obtained from Dr. L. Montagnier (at that time at the Pasteur Institute, Paris, France).

### Compounds

The sources of the compounds were as follows: acyclovir [ACV, 9-(2-hydroxyethoxymethyl)-guanine], GlaxoSmithKline, Stevenage, UK; PMEA [adefovir, 9-[2-(phosphonylmethoxyethyl)-adenine]; and (*R*)-PMPA [tenofovir, (*R*)-9-[2-(phosphonylmethoxypropyl)adenine]], Gilead Sciences, Foster City, CA. PMEO-DAPym was synthesized by A. Holý (Prague, Czech Republic) [46], [47]. PMEO-DAPym was also synthesized and provided by Shanghai Medicilon Inc., Shanghai, China. Tenofovir diphosphate (tenofovir-DP) and adefovir diphosphate (adefovir-DP) were obtained from Moravek Biochemicals, Brea, CA, and the diphosphate of PMEO-DAPym was synthesized by A. Holý, Prague, Czech Republic.

The stock solutions of PMEA, PMPA, and PMEO-DAPym (10 mg/ml) were tested for endotoxin content with the Limulus Amebocyte Lysate assay (Cambrex Bioscience, Verviers, Belgium) and were found to contain <1 ng/ml endotoxin.

### HSV cytopathicity measurements

The HSV-induced cytopathic effect (CPE) was evaluated in HSV-infected HEL and PHK cultures as described [44], [48]. Briefly, cells were infected with each viral strain at 100 tissue culture infective dose-50% (TCID_50_) (1 TCID_50_ being the 50% tissue culture infective dose, or virus dose required to infect 50% of the virus-exposed cell cultures) and cultured in 96-well microtiter plates for 3 days in the presence of several concentrations of the test compounds. After the incubation period at 37°C, CPE was visually assessed, and the 50% effective concentration (EC_50_, the compound concentration required to reduce viral CPE by 50%) was determined.

### Virus yield reduction assays

These assays were carried out in HEL cell monolayers at different times post infection. Cells were grown in 24-well microtiter plates and infected with one HSV-1 (RV-174) or two HSV-2 clinical isolates (NS and RV-124) at the indicated multiplicity of infection (m.o.i.). After 2 h at 37°C, the cells were washed and medium containing different concentrations of PMEO-DAPym (in duplicate) was added. Following 24, 48, and 72 h of incubation, we released the viruses by freeze-thawing and then titrated them using a plaque assay in HEL cells. The EC_90_ and EC_99_ are defined as the drug concentrations causing a 90% (one order of magnitude) or 99% (two orders of magnitude) reduction, respectively, in virus production as measured following viral titration by plaque assay.

### Herpesvirus infection of primary monocyte/macrophage cell cultures

We obtained human PBMC from the blood of healthy seronegative donors using Ficoll–Hypaque density gradient centrifugation. The PBMC were resuspended in RPMI 1640 medium supplemented with 20% heat-inactivated serum and then seeded into 48-well plates (1.8×10^6^ cells/well). M/M were separated by adherence onto plastic. After 5 days, non-adherent cells were carefully removed by repeated gentle washings with warm medium, and adherent M/M were cultured for an additional 3 days to mature and to form a monolayer. M/M were estimated to be 10^5^ cells/well at the time of infection. To evaluate the anti-HSV-2 activity of PMEO-DAPym on human macrophages, we added the compound to macrophages 1 h before infection at a variety of concentrations (0.0032, 0.016, 0.08, 0.4, 2, 10, or 50 µg/ml). Similarly, macrophage cultures were treated with the same concentrations of adefovir and tenofovir. Macrophage cultures were then infected with HSV-2 (G) (100 TCID_50_) in the presence of the compounds. After 2 h of adsorption, the cultures were extensively washed to remove any residual virus particles. Fresh complete medium and compounds, at the established concentrations, were then added to the cultures. Appropriate positive (infected but not treated) and mock-infected negative (uninfected and untreated) M/M controls were run for each experiment as well. All assays were performed in triplicate. The HSV-2-induced cytopathic effect was found to be complete 120 h after virus challenge of infected but untreated M/M [49]. We determined the amount of infectious virus in the supernatants 5–6 days after infection using the cytopathic effect assay. Tenfold serially diluted supernatants were added to confluent monolayers of Vero cells in 96-well plates (100 µl/well, 6 parallel wells). The plates were incubated at 37°C for 4 or 5 days, at which time the cytopathic effect was demonstrated. The titers of virus produced were calculated according to the Reed and Muench method and expressed as 50% tissue culture infective dose per mL (TCID_50_/mL).

### Organotypic epithelial raft cultures

Primary human keratinocytes (PHKs) were seeded on top of collagen gels in 24-well microtiter plates and maintained submerged for 24–48 h. The collagen rafts were then raised and placed into stainless-steel grids at the interface between air and the liquid culture medium. The epithelial cells were allowed to stratify. Rafts were infected with 5,000 PFU of HSV-1_KOS_ or HSV-2_G_ at 10 days post lifting and treated with adefovir or PMEO-DAPym. Five days later, one series of rafts was fixed in 10% buffered formalin, embedded in paraffin, and stained with hematoxylin and eosin for histological evaluation. Another series of rafts was used to quantify virus production. For that purpose, each raft was frozen in 3 ml of phosphate buffered saline (PBS) and thawed to release the virus from the infected epithelium. We clarified supernatants by centrifugation at 1,800 rpm and titrated them using a plaque assay in HEL cells. Titers were calculated as plaque forming units (PFU) per ml of virus suspension. Virus production per raft was then calculated. Two rafts per drug concentration were used to determine the effects of the compounds on virus yield.

### Human ex vivo tissues

Human tonsillar tissues were obtained from patients undergoing routine tonsillectomy at the Children's National Medical Center (Washington, DC) under IRB-approved protocol. Cervical tissues were obtained through the National Disease Research Interchange (NDRI, Philadelphia, PA). Tissues were dissected into blocks of about 2×2×2 mm and placed onto collagen sponge gels in culture medium at the air-liquid interface as described earlier [24]. For tonsillar tissue for each experimental condition, 27 tissue blocks (9 blocks/well filled with 3 ml of medium) were inoculated with HSV-1 (strain F) or HSV-2 (strains G and MS). In coinfection experiments, tissue blocks were sequentially infected with HSV-2 (G) and HIV-1 (III_B_) (obtained from the Rush University Virology Quality Assurance Laboratory, Chicago, IL). Drugs (adefovir and PMEO-DAPym) were added to the culture medium 12 h prior to viral infection and replenished at each culture medium change.

For cervico-vaginal tissue, 16 blocks were infected by immersion in HSV-2_G_-containing medium and maintained on the gelfoam rafts. Adefovir was added at the time of infection and replenished at each culture medium change. HSV replication was evaluated from the release of viral DNA into the culture medium as measured with quantitative real-time PCR [18]. We evaluated HIV-1 replication from the release of p24 capsid antigen using a bead-based assay [50].

### In vivo antiviral activity in HSV-1- and HSV-2-infected mice

Female adult NMRI athymic nude mice or hairless mice (weighing ∼20 g and approximately 4 weeks old) were scarified on the lumbosacral area over a surface of about 1 cm^2^ with 5×10^3^ PFU of HSV-1 (KOS) or 5×10^2^ PFU of HSV-2 (G).

Formulations of adefovir and PMEO-DAPym (0.3% and 1%) in a gel (identical to that used in the CAPRISA 004 trial) were applied topically twice a day for a period of 5 days starting 1–2 h post infection. In each experiment, a group of animals was treated with a placebo formulation that contained exactly the same vehicle but without drug. All animal procedures were approved by the KU Leuven Animal Care Committee. Development of lesions and mortality were recorded over a 1-month period. We estimated survival rates according to the Kaplan-Meier method and compared them using the log-rank test (Mantel-Cox) with GraphPad Prism. Animals' care was in accordance with institutional guidelines and the ethical committee of the KU Leuven.

### HSV-1 DNA polymerase and HIV-1 reverse transcriptase assay

The reaction mixture (40 µl) for the HSV-1 DNA polymerase and HIV-1 RT assays contained 4 µl of Premix (200 mM Tris.HCl, pH 7.5; 2 mM DTT; 30 mM MgCl_2_), 4 µl of BSA (5 mg/ml), 1.6 µl of activated calf thymus DNA (1.25 mg/ml), 0.8 µl of dCTP (5 mM), 0.8 µl of dTTP (5 mM), 0.8 µl of dGTP (5 mM), 2 µl of radiolabeled [^3^H]dATP (1 mCi/ml) (3.2 µM), 18 µl of H_2_O, and 4 µl of adefovir-pp or PMEO-DAPym-pp at different concentrations (i.e., 200, 20, 2, and 0.2 µM). In the HSV DNA polymerase assays, the inhibitory effect of adefovir-pp or PMEA-DAPym-pp on herpesvirus DNA polymerase-catalyzed polymerization was also determined in the presence of radiolabeled [^3^H]dGTP (1 mCi/ml; 2.8 µM), [^3^H]dTTP (1 mCi/ml; 1 µM), or [^3^H]dCTP (1 mCi/ml; 2.5 µM) as competing dNTP in the presence of 0.8 µl (5 mM) of the other dNTPs in the reaction mixture as described above. The reaction was started by the addition of 4 µl of recombinant HSV-1 DNA polymerase (kindly provided by M.W. Wathen, at that time at Pfizer, Kalamazoo, MI) or 4 µl of recombinant HIV-1 RT (in 20 mM Tris.HCl, pH 8.0; 1 mM DTT; 0.1 mM EDTA; 0.2 M NaCl; 40% glycerol), and the reaction mixture was incubated for 60 min (HSV-1 DNA polymerase) or 30 min (HIV-1 RT) at 37°C. Then, 1 ml of ice-cold 5% TCA in 0.02 M Na_4_P_2_O_7_.10 H_2_O was added to terminate the polymerisation reaction, after which the acid-insoluble precipitate (radiolabeled DNA) was captured onto Whatman glass fiber filters (type GF/C; GE Healthcare UK Limited, Buckinghamshire, UK) and further washed with 5% TCA and ethanol to remove free radiolabeled dNTP. Radioactivity was determined in a Perkin Elmer Tri-Carb 2810 TR liquid scintillation counter.

### Flow cytometric analyses

PBMC were cultured in the presence of adefovir, tenofovir, and PMEO-DAPym and incubated at 37°C in a humidified, 5% CO_2_-controlled atmosphere. The expression of cellular activation markers was measured after 3 days of culture. Briefly, after washing the cells with PBS containing 2% FCS, we incubated them with PerCP-conjugated anti-CD4 mAb (clone SK3) in combination with PE-conjugated anti-CD25, anti-CD69, or anti-HLA-DR mAbs for 30 min at room temperature. For aspecific background staining, cells were stained in parallel with Simultest Control IgG γ1/γ2a and PerCP-conjugated mouse IgG1. Finally, the cells were washed with PBS, fixed with 1% formaldehyde solution, and analyzed with a FACSCalibur (BD Biosciences, San Jose, CA); data were acquired with CellQuest software and further analyzed with the FLOWJO software (Tree Star, San Carlos, CA). The expression of the chemokine receptors was measured after 24 h, and PBMC were incubated with PerCP-conjugated anti-CD4 mAb (clone SK3) in combination with APC-conjugated anti-CXCR4 mAb (clone 12G5) and PE-conjugated anti-CCR5 (clone 2D7) or anti-CXCR3 (clone 1C6). To evaluate the biological effects of the β-chemokines produced by drug-treated PBMC, freshly isolated PBMC were incubated for 1 h at 37°C with supernatants collected from PBMC cultures treated with compounds for 24 h. Then, cells were incubated with PerCP-conjugated anti-CD4 mAb (clone SK3) in combination with PE-conjugated anti-CCR5 mAb (clone 2D7) for 30 min at room temperature. As a control, cells were also incubated for 1 h at 37°C with the LD78β isoform of MIP-1 alpha (PeproTech, London, United Kingdom), which is described as potently downregulating the CCR5 receptor [51]. All mAbs were purchased from BD Biosciences (Erembodegem, Belgium).

### Bio-Plex chemokine assay

Freshly isolated PBMC were cultured in the presence of adefovir, tenofovir, and PMEO-DAPym, and culture supernatants were collected after 24 h. We determined the cytokine production profile using the Bio-Plex 200 system (Bio-Rad, Hercules, CA) and a Bio-Plex Human chemokine assay according to the manufacturer's instructions and as described in detail earlier [52]. The assay kit contains beads conjugated with mAbs specific for interferon-inducible protein-10 (IP-10), macrophage inflammatory protein-1α (MIP-α), and MIP-1β, and regulated on activation normal T-cell expressed and secreted (RANTES). For each chemokine, eight standards ranging from approximately 1.5 pg/ml to 32,000 pg/ml were constructed, and the minimum detectable dose was between 1.5 pg/ml and 8 pg/ml. Standard curves and the concentrations of chemokines within samples were generated with the Bio-Plex Manager 4.1 software.

### Detection of chemokine mRNA

We isolated total RNA from approximately 10^6^ PBMC using the RNeasy Mini Kit (QIAGEN, Hilden, Germany). To eliminate potential genomic DNA contamination, the samples were treated with DNase I (Roche). Total RNA (300 ng) was reverse-transcribed to cDNA by means of Moloney murine leukemia virus reverse transcriptase (Invitrogen) and Hexamer Primer (Invitrogen) according to the manufacturer's instructions.

For RT-PCR, the primers used were 5′-GCAACCAGTTCTCTGCATCA-3′ (sense) and 5′-TTCTGGACCCACTCCTCACT-3′ (antisense) for human CCL3/MIP-1α; 5′-AAGCTCTGCGTGACTGTCCT-3′ (sense) and 5′-CCAGGATTCACTGGGATCAG-3′ (antisense) for human CCL4/MIP-1β; 5′-CGCTGTCATCCTCATTGCTA-3′ (sense) and 5′-ACTCCCGAACCCATTTCTTC-3′ (antisense) for human CCL5/RANTES; 5′-ATCCTCACCCTGAAGTACCCCA-3′ (sense) and 5′-GAAGGTCTCAAACATGATCTGGGT-3′ (antisense) for human β-actin; and 5′-CGAGATCCCTCCAAAATCAA-3′ (sense) and 5′-ACAGTCTTCTGGGTGGCAGT-3′ (antisense) for human glyceraldehyde-3-phosphate dehydrogenase (GAPDH). The reaction mixtures contained dNTPs (Invitrogen) at 100 nM, each of the forward and reverse primers at 0.5 µM, and 0.5 U of SuperTaq DNA polymerase (HT Biotechnology, Cambridge, UK) in a total volume of 50 µl. After electrophoresis through a 2% agarose gel, the amplified cDNA fragments were visualized with ethidium bromide.

## Supporting Information

Figure S1 **Overview of the cytokine profiles of PBMC incubated for 24 h with medium only or with 500 µg/ml of PMEO-DAPym, adefovir or tenofovir.** Supernatants from PBMC cultures derived from healthy donors were collected and cytokine levels were measured by the Bio-Plex array system.
(TIF)

Figure S2 **Overview of the cytokine profiles of PHA-stimulated PBMC incubated for 24 h with medium only or with 500 µg/ml of PMEO-DAPym, adefovir or tenofovir.** Supernatants from PHA-stimulated PBMC cultures derived from healthy donors were collected and cytokine levels were measured by the Bio-Plex array system.
(TIF)
