# Supplementary figures and images for: A Multi-targeted Drug Candidate with Dual Anti-HIV and Anti-HSV Activity
Source: PLoS Pathog. 2013 Jul 25;9(7):e1003456. doi: 10.1371/journal.ppat.1003456 (PMC3723632; doi:10.1371/journal.ppat.1003456)

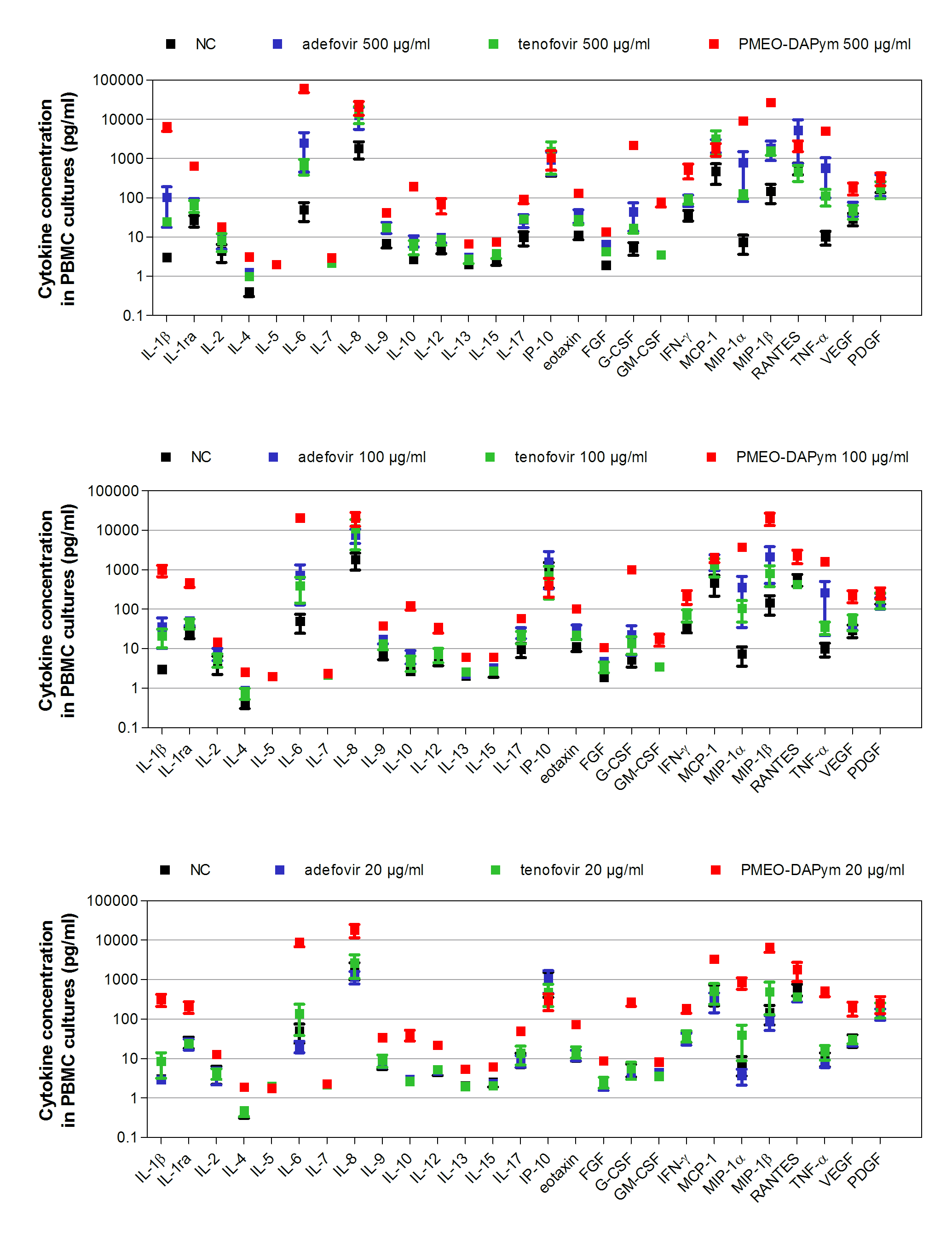

Supplement: Figure S1 — Overview of the cytokine profiles of PBMC incubated for 24 h with medium only or with 500 µg/ml of PMEO-DAPym, adefovir or tenofovir. Supernatants from PBMC cultures derived from healthy donors were collected and cytokine levels were measured by the Bio-Plex array system. (TIF) [file ppat.1003456.s001.tif]

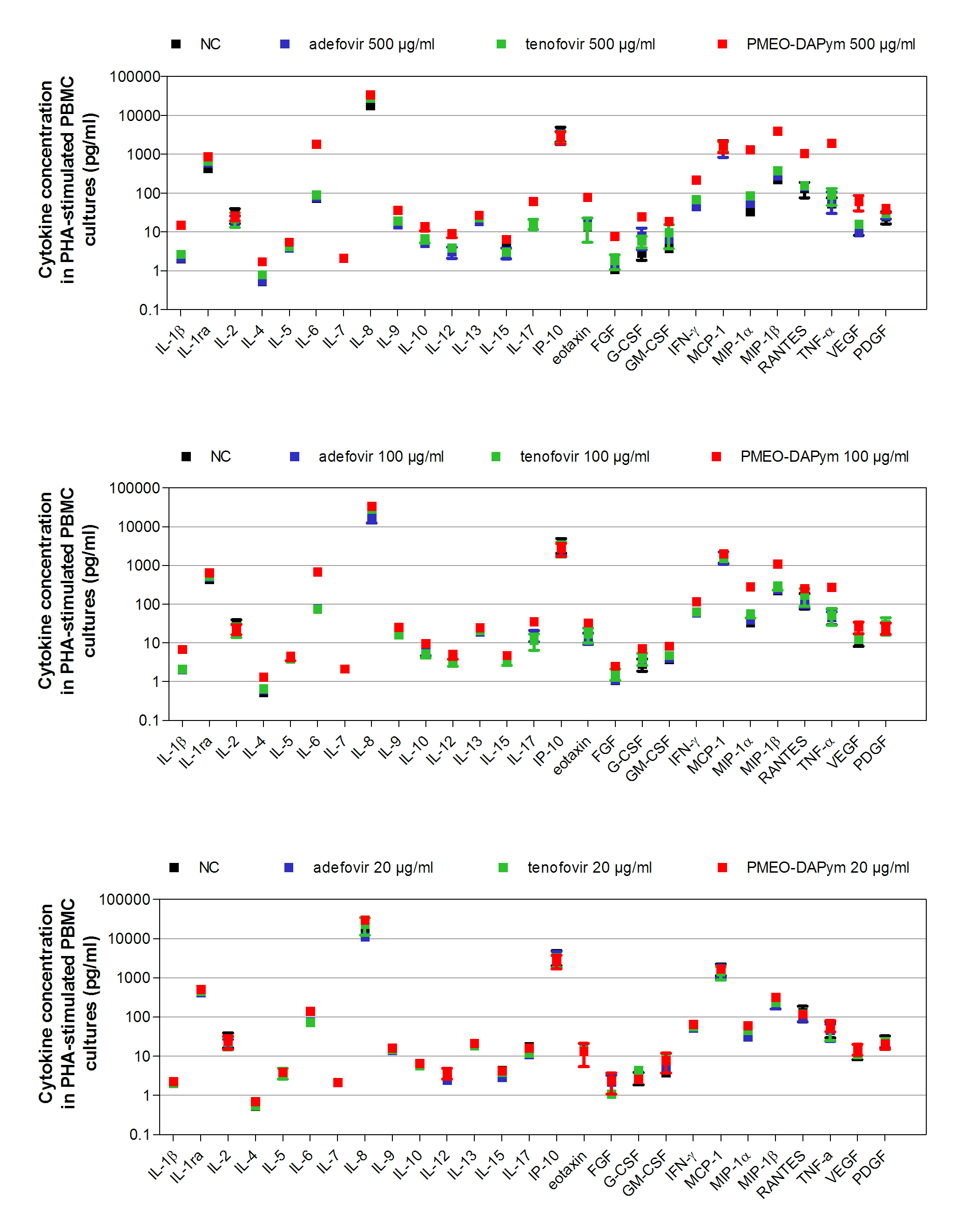

Supplement: Figure S2 — Overview of the cytokine profiles of PHA-stimulated PBMC incubated for 24 h with medium only or with 500 µg/ml of PMEO-DAPym, adefovir or tenofovir. Supernatants from PHA-stimulated PBMC cultures derived from healthy donors were collected and cytokine levels were measured by the Bio-Plex array system. (TIF) [file ppat.1003456.s002.tif]
